# Reduning injection combined with western medicine for pneumonia: A protocol for systematic review and meta-analysis

**DOI:** 10.1097/MD.0000000000022757

**Published:** 2020-10-23

**Authors:** Chenggang Cao, Zelong Zhen, Shengnan Kuang, Tao Xu

**Affiliations:** a Chongqing Rongchang District People's Hospital; b Chongqing Fifth People's Hospital, Chongqing, Chongqing Municipality, China.

**Keywords:** meta-analysis, pneumonia, protocol, Reduning injection

## Abstract

**Background:**

Pneumonia is a common respiratory disease. In severe cases, it can induce cardiovascular disease and even life-threatening. In particular, pneumonia caused by the new coronavirus (SARS-CoV-2) that broke out at the end of 2019 has seriously affected the health of people in all countries. In recent years, it has been treated with the combination of traditional Chinese medicine (TCM) (such as Reduning injection) and Western medicine, and its mortality has decreased significantly. But their efficacy has not been scientifically and systematically assessed. Accordingly, it is essential to provide a systematized review program to estimate the efficacy and safety of Reduning injection combined with Western medicine to treat pneumonia.

**Methods:**

The following databases are retrieved from start to September 2020: Pubmed, Cochrane Library, EMBASE, Web of Science, Chinese National Knowledge Infrastructure (CNKI), Wanfang database, the Chongqing VIP Chinese Science and Technology Periodical Database (VIP) databases, Chinese Biomedical Literature Database (CBM), and other databases, which are absorbed into clinical RCTs of pneumonia using western medicine alone or plus Reduning injections. The selection of studies, data extraction, and assessment of risk of bias will be performed independently by 2 reviewers. At the same time, Review Manager V.5.3.5 (Rev Man V.5.3.5) was used for bias risk assessment and data synthesis.

**Results:**

The efficacy and safety of Reduning injection combined with western medicine in the treatment of pneumonia were evaluated in terms of overall effective rate, the patient's antipyretic time, antitussive time, rales disappearing time, X-ray recovery time, and the incidence of adverse reactions.

**Conclusions:**

This study provides reliable evidence-based support for the clinical application of Reduning injection combined with western medicine for pneumonia.

**Ethics and dissemination:**

Ethical approval is not required in this secondary research evidence, and we will publish the results of this study in a journal or relevant conferences.

**Registration number:**

DOI 10.17605/OSF.IO/VS75Y.

## 1 Introduction

Respiratory infections are a chief reason of infectious disease death all over the world.^[1,2]^ The most common definition of pneumonia is an infection of all the parts of the lungs associated with gas transfer, namely lung parenchyma, respiratory bronchioles, alveolar ducts, and alveoli. There are 50% of intensive care units (ICUs) patients all over the world, who have acute pulmonary infections.^[3,4]^ The cause of pneumonia is more common with viral infection and bacterial infection.^[4]^ The new coronavirus pneumonia (COVID-19) that broke out at the end of 2019 is caused by the new coronavirus (SARS-CoV-2).^[6–8]^ As an acute respiratory infectious disease, COVID-19 has been included in Category B infectious diseases as stipulated in the Law of the People's Republic of China on the Prevention and Control of Infectious Diseases, which is listed as Category A infectious diseases.^[6–8]^

In recent years, it has been treated with the combination of TCM (such as Reduning injection) and Western medicine, and its mortality has decreased significantly.^[5,9–11]^ TCM injection is the main type of TCM for syndrome differentiation of TCM therapy, but its effect is relatively slow.^[10,11]^ Although Western medicine routine treatment has quick effect and remarkable effect, it also has shortcomings such as drug resistance and is not suitable for long-term use.^[11]^ Reduning injection has achieved a certain effect in clinical treatment of acute bronchitis and upper respiratory tract infection.^[5,9–11]^ In the course of the outbreak of COVID-19, Reduning injection is widely used to treat severe patients with COVID-19 in China, which is also recommended for use in *the Guideline on Diagnosis and Treatment of Coronavirus Disease 2019* (Revised 7th version) officially issued by National Health Commission of the People's Republic of China.^[6–8]^ Western medicine routine treatment mainly uses antibiotics (penicillins, cephalosporins, macrolides, and aminoglycosides etc.)^[12–21]^ and antiviral drugs (interferon and ribavirin).^[22–25]^ However, the evaluation of their efficacy is not scientific and systematic.

Accordingly, it is essential to provide a systematized review program to estimate efficacy and safety of Reduning injection combined with Western medicine to treat pneumonia.

## 2 Methods

### 2.1 Protocol register

This protocol of systematic review and meta-analysis has been drafted under the guidance of the preferred reporting items for systematic reviews and meta-analyses protocols (PRISMA-P). Moreover, it has been registered on open science framework (OSF) on September 8, 2020. (Registration number: DOI 10.17605/OSF.IO/VS75Y).

Any changes in the standard protocol will be described further.

### 2.2 Ethics and dissemination

Since this study is a secondary analysis of existing literature, ethical approval is not required. We will provide a systematical view and evidence of Reduning injection combined with western medicine for pneumonia, which will benefit clinical practice and further research. Also, we will publish our study in a peer-reviewed journal or distributed at related conferences.

### 2.3 Inclusion criteria

#### 2.3.1 Types of studies

We are eligible for a RCTs of Reduning injection combined with Western medicine to treat pneumonia.

#### 2.3.2 Types of participants

According to the international or Chinese official published diagnostic criteria for pneumonia, patients with a definite diagnosis of pneumonia are included with no restriction on nationality, region, race, gender, age, course of disease, or type of pneumonia.

#### 2.3.3 Intervention measures

The experimental intervention group is treated with Reduning injection combined with Western medicine. The control group is treated with conventional Western medicine. Conventional Western medicine treatment is mainly based on antibiotics (penicillins, cephalosporins, macrolides, etc.) and antiviral drugs (interferon and ribavirin). We excluded RCTs comparing simple Reduning injection with Western medicine.

#### 2.3.4 Outcome indicators

##### 2.3.4.1 Primary outcome

Overall effective rate = (number of cured + number of marked effect) / total number of people. The evaluation criteria of efficacy are:^[26]^ Recovery: the body temperature returned to normal, and respiratory tract symptoms such as cough and sputum were completely or basically relieved. The X-ray chest radiographs showed that the lung shadow was small or not expanding, and leukocyte or neutrophil examination was close to normal. Significant effect: the body temperature was basically normal, and the respiratory symptoms such as cough and sputum had improved. There was no enlargement of lung shadows in the X-ray chest radiographs and no increase in blood leukocytes or neutrophils. Invalid: fever persisted, and respiratory tract symptoms such as cough and sputum did not change or worsened. The X-ray chest radiograph showed enlarged or new lung shadows, and the white blood cells are elevated or persistently lower than normal.

##### 2.3.4.2 Secondary outcomes

Include the patient's antipyretic time, antitussive time, rales disappearing time, X-ray recovery time, and the incidence of adverse reactions (such as allergic reactions, gastrointestinal reactions, blood system reactions and pyrogenic reactions, etc.)

### 2.4 Exclusion criteria

1. If the paper is repeatedly published, the one with the most complete data shall be included;
2. Articles with missing data and we are still unable to obtain data after contacting the author;
3. Articles with obvious data errors;
4. Low-quality literature will be excluded, and low-quality reference standard is the standard in Jiaguo Zhaos paper.^[27]^ The included literature was evaluated as low, medium, and high quality according to the following criteria: ① If randomization or allocation-hiding assessments were high risk, the study was rated as low quality regardless of other risks; ② When the randomization and allocation of hidden assessment is low risk, and all other quality assessment are assessed as low risk or clear, the study is rated as high quality; ③ If the research is neither in line with high quality, nor in line with low quality standards, it is considered to be of medium quality research.

### 2.5 Search strategy

#### 2.5.1 Electronic searches

These databases will be retrieved from start to May 2020: Pubmed, Cochrane Library, EMBASE, Web of Science, CNKI, Wanfang database, VIP databases, and CBM. In order to comprehensively obtain RCTs of Reduning injection combined with Western medicine for pneumonia, we searched baidu academic, Google academic, as well as the International Clinical Trials Registry Platform (ICTRP), Chinese Clinical Trial Registry and Clinical Trials to obtain grey literature and relevant data that have not been publicly published. These are only trials report including English and Chinese.

#### 2.5.2 Searching strategy

Table 1 shows the PubMed search method. Other electronic databases also use the method described above.

**Table 1:**
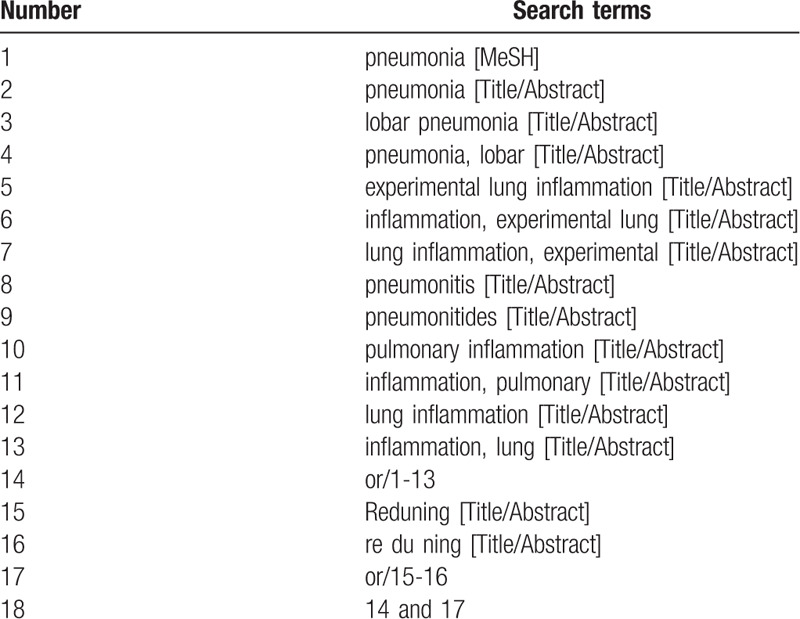
Search strategy in PubMed database.

#### 2.5.3 Selection process

**Figure 1:**
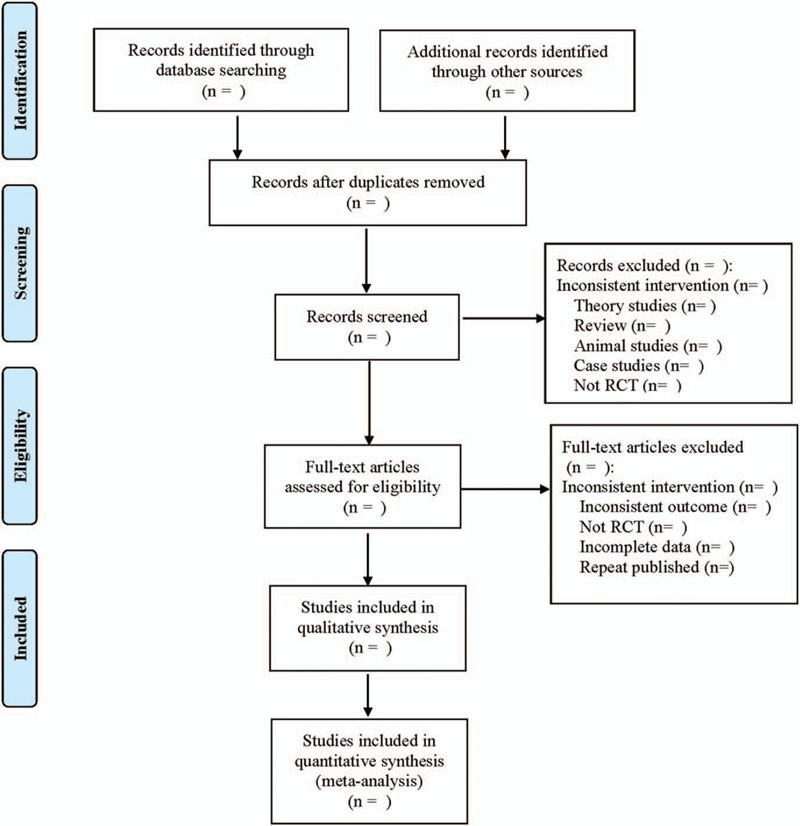
Flow diagram.

This research screening will be performed by 2 independent reviewers. Read titles, abstracts and full-texts of research, and evaluate whether it meets the requirements according to the inclusion criteria. The third reviewer will decide several studies with different opinions. The research screening process is defined for a systematic review and meta-analysis flow diagram (Fig. 1).

### 2.6 Data screening and extraction

Referring to the method of research selection in version 5.0 of the Cochrane collaboration Network system Evaluator Manual, according to the PRISMA flow chart, the 2 researchers used the EndNote X9 document management software to independently screen and check the literature according to the above inclusion and exclusion criteria, and check each other, if there were different opinions, negotiate with a third party to resolve the differences. At the same time, Excel 2013 was used to extract relevant information, including: ① basic information of the included study, including research title, author name, and publication year, etc.; ② baseline characteristics of the included study, including sample size, patient age, sex ratio, and course of disease of the treatment group and control group; ③ intervention methods, intervention time, frequency, dose and route of the treatment group and control group; ④ the key elements of bias risk assessment; ⑤ the outcome indicators concerned and the relevant measurement data.

### 2.7 Literature quality assessment

The assessment of risk of bias will be carried out independently by 2 reviewers via the Cochrane Collaboration's “Risk of bias” tool. According to these criteria (random sequence generation, allocation concealment, blinding, incomplete data, selective result reports and other bias), study bias is classified into the following levels: unclear, low, and high risk. If the assessment of bias causes controversy, it will be necessary to discuss it with the third reviewer. The RevMan V.5.3.5 is applied to study graphic representations of potential bias within and throughout research.

### 2.8 Statistical analysis

#### 2.8.1 Data analysis and processing

Statistical analysis is performed by a 95% CIs. Odds ratio (OR) and relative risk (RR) are usually applied to dichotomy result data. For continuous results, if the measurement method and measurement unit are the same, the weighted mean difference (WMD) shall be used; if the measurement method or measurement unit is different, the standard mean difference (SMD) shall be used for statistical analysis. Based on the Cochrane Handbook, χ^2^ test, *I*^*2*^ value of forest and plot is measured to evaluate heterogeneity. *I*^*2*^ ≥ 50% is regarded as a measure of severe heterogeneity, and *I*^*2*^ < 50% is considered that there is no heterogeneity. Therefore, the conclusion is drawn as appropriate. If there is no clinical and methodological heterogeneity, the random effect model will be evaluated by merger analysis. If the heterogeneity is oversized, a descriptive analysis will be conducted. Based on sample size of the clinical study, including measurement, intervention methods and the length of treatment, we will conduct a systematized review of the literature through meta-analysis. A meta-analysis including multiple homogeneity studies will be performed by Rev Man V.5.3.5. When *I*^*2*^ < 50%, the fixed effect model will be chosen; When *I*^*2*^ ≥ 50%, the sources of heterogeneity are analyzed, and subgroup analysis may be performed. If there is statistical heterogeneity between studies but no clinical heterogeneity, a random effect model will be selected to analyze. Otherwise, we will exclude the research from meta-analysis.

#### 2.8.2 Dealing with missing data

When data for some research results are insufficient or missing, we will contact the corresponding author via e-mail or phone to supplement their content. Articles that do not provide data or are unable to calculate the given information will be excluded.

#### 2.8.3 Unit of analysis issues

The units of each outcome from different studies will be converted to the International System of Units before statistical analysis.

#### 2.8.4 Assessment of publication bias

Funnel plots and statistic tests will be used to detect overall estimated reporting bias. Eggers and Beggs test are used to quantitatively assess potential publication bias.

#### 2.8.5 Subgroup analysis and investigation of heterogeneity

Subgroup analysis will be carried out to evaluate heterogeneity. The various types (bacterial pneumonia, mycoplasma pneumonia, common viral pneumonia, COVID-19, etc), severity of pneumonia (mild pneumonia, common pneumonia, severe of pneumonia), age of patients (pediatric pneumonia, adult pneumonia, elderly pneumonia), and course of treatment may have an effect on heterogeneity.

#### 2.8.6 Sensitivity analysis

Sensitivity analysis will be applied to test the robustness of key decisions made during the review process. The sensitivity analysis of all indicators is carried out through one-to-one elimination method to verify the stability of the obtained results by Rev Man V.5.3.5.

#### 2.8.7 Evidence quality evaluation

The Grading of Recommendations Assessment, Development, and Evaluation (GRADE) will be used to assess the quality of evidence. It contains 5 domains (bias risk, consistency, directness, precision, and publication bias). And the quality of evidence will be rated as high, moderate, low, and very low.

## 3 Discussion

The main pathogenic characteristics of pneumonia are acute onset, severe symptoms, and many complications, which can easily cause circulatory system failure and seriously threaten peoples health.^[1–4]^ At the same time, patients with pneumonia may have varying degrees of fever, cough, shortness of breath, and irritability, whose lungs exist wet rales and have solid tissue changes. In addition, their X-ray film shows a flaky shadow and serum IgM antibody is strongly positive.^[1–4]^ In recent years, it has been treated with the combination of TCM (such as Reduning injection) and Western medicine, and its mortality has decreased significantly. Reduning injection is mainly composed of Artemisia annua, honeysuckle, and Gardenia jasminoides Ellis. It is mainly used in the upper respiratory tract infection (exogenous wind heat syndrome) caused by high fever, slight evil wind cold, head and body pain, cough, phlegm yellow, and other diseases.^[5,9–11]^ Previous pharmacological studies have shown that paraquat modulates AMPK/MAPK/NF-κB signaling to reduce acute pulmonary injury. In the treatment of COVID-19,^[28]^ Reduning injection may be associated with anti-inflammatory and immunoregulation effects of the active ingredients via multiple targets and pathways.^[6–8,29]^ Western medicine routine treatment is mainly based on antibiotics and antiviral drugs, such as penicillin, ceftriaxone sodium, amoxicillin, amoxicillin and clavulanate, erythromycin, azithromycin, minocycline, doxycycline, clarithromycin, levofloxacin, interferon, ribavirin, oseltamivir, ganciclovir, and so on.^[12–25]^ There are many Western medicines used in clinical. Therefore, the systematic review assesses efficacy and safety of Reduning injection combined with Western medicine to treat pneumonia and provide evidence to clinicians.

However, this systematic review has some limitations. For example, diagnose criteria, dosage, duration of Reduning Injection, and no blind method in the trials may result in insignificant heterogeneity. Research published in English and Chinese are retrieved. There is no related research in other languages.

## Author contributions

**Data curation:** Chenggang Cao, Zelong Zhen.

**Funding acquisition:** Tao Xu.

**Investigation:** Tao Xu.

**Resources:** Chenggang Cao.

**Software:** Zelong Zhen, Shengnan Kuang.

**Writing – original draft:** Chenggang Cao, Zelong Zhen.

**Writing – review & editing:** Tao Xu.
